# Children and adolescents referred for treatment of anxiety disorders: Differences in clinical characteristics

**DOI:** 10.1016/j.jad.2014.06.028

**Published:** 2014-10-01

**Authors:** Polly Waite, Cathy Creswell

**Affiliations:** School of Psychology and Clinical Language Sciences, Whiteknights, University of Reading, Reading RG6 6AL, UK

**Keywords:** Anxiety, Childhood, Adolescence, Diagnoses, Comorbidities

## Abstract

**Background:**

Reports of the clinical characteristics of children and adolescents with anxiety disorders are typically based on community populations or from clinical samples with exclusion criterion applied. Little is known about the clinical characteristics of children and adolescents routinely referred for treatment for anxiety disorders. Furthermore, children and adolescents are typically treated as one homogeneous group although they may differ in ways that are clinically meaningful.

**Methods:**

A consecutive series of children (*n*=100, aged 6–12 years) and adolescents (*n*=100, aged 13–18 years), referred to a routine clinical service, were assessed for anxiety and comorbid disorders, school refusal and parental symptoms of psychopathology.

**Results:**

Children with a primary anxiety disorder were significantly more likely to be diagnosed with separation anxiety disorder than adolescents. Adolescents with a primary anxiety disorder had significantly higher self and clinician rated anxiety symptoms and had more frequent primary diagnoses of social anxiety disorder, diagnoses and symptoms of mood disorders, and irregular school attendance.

**Limitations:**

Childhood and adolescence were considered categorically as distinct, developmental periods; in reality changes would be unlikely to occur in such a discrete manner.

**Conclusions:**

The finding that children and adolescents with anxiety disorders have distinct clinical characteristics has clear implications for treatment. Simply adapting treatments designed for children to make the materials more ‘adolescent-friendly’ is unlikely to sufficiently meet the needs of adolescents.

## Highlights

• Children and adolescents with anxiety disorders have distinct characteristics.
• Children have more diagnoses of separation anxiety disorder than adolescents.
• Adolescents have more primary diagnoses of social anxiety disorder than children.
• Adolescents have more severe symptoms of anxiety than children.
• Adolescents have more comorbid mood disorders and school refusal than children.

## 1 Introduction

Anxiety disorders typically have an early age of onset, with a mean of 10 years or younger (Keller et al., 1992, Orvaschel et al., 1995), and are among the most common psychiatric disorders experienced by children and adolescents (Essau and Gabbidon, 2013). They cause substantial impairment at school, home and socially (Essau et al., 2000, Wittchen et al., 1999) and, without treatment, often continue into adulthood and are associated with negative life course outcomes (Last et al., 1997, Pine et al., 1998). Consequently, it is of great importance to have a clear understanding of how anxiety disorders present in children and adolescents seeking treatment, in order to develop and refine effective treatments.

Randomized controlled trials of psychological treatments (most commonly Cognitive Behavior Therapy, CBT) for anxiety disorders in youth have typically included children and adolescents from a broad age range (e.g. 7–17 years, Walkup et al., 2009) and treatment programs have often been developed with samples that are predominantly in middle childhood (e.g. Barrett et al., 1996, Kendall, 1994), with very little theory or practice-research focusing on adolescents with anxiety disorders (Kendall et al., 2013). A first step in establishing whether and how treatments may need to be adapted for adolescents referred for treatment for anxiety disorders is determining whether their clinical characteristics differ from their younger counterparts.

Understanding of the epidemiology of anxiety disorders in childhood and adolescence has been informed by a number of large community studies. The majority of studies have found that differences in the prevalence of particular anxiety disorders emerge as children move into adolescence, with decreased rates of separation anxiety disorder (SAD) (Cohen et al., 1993, Compton et al., 2000, Copeland et al., 2014, Costello, 2003) and increased rates of panic disorder, agoraphobia and obsessive compulsive disorder (OCD) among both sexes (Costello et al., 2003, Ford et al., 2003), and social anxiety disorder and GAD in girls (Copeland et al., 2014, Costello et al., 2003). There is also some evidence for a sharp decrease in anxiety disorders generally around the age of 11 to 12 years, as rates of SAD decline, before overall rates increase across disorders from early adolescence to young adulthood (Copeland et al., 2014).

Most reports of the clinical characteristics of anxiety disorders include children and adolescents from a broad age range, e.g. 5–18 years (Last et al., 1992), 7–17 years (Kendall et al., 2010) and there has been relatively little examination of whether differences apply between clinical populations of children and adolescents referred for treatment of anxiety disorders. When children and adolescents seeking help for anxiety disorders have been compared the pattern of results is broadly consistent with community studies, with children having more symptoms and more frequent diagnoses of SAD and adolescents having more social anxiety symptoms/diagnoses (Compton et al., 2000, Esbjørn et al., 2010, Kendall et al., 2010). Indeed in a Danish sample of referred children and adolescents (Esbjørn et al., 2010), the frequency of social anxiety disorder diagnoses was over five times higher in adolescent boys, and over 10 times higher in adolescent girls, compared to the rates in children. Other differences in clinical characteristics between children and adolescents with anxiety disorders include greater comorbidity among children and higher clinician-rated severity among adolescents (in a research population with primary diagnoses of SAD, social anxiety disorder or generalized anxiety disorder (GAD) (Kendall et al., 2010). These findings are important as elevated initial severity, the presence of social anxiety disorder and older age (7–11 years versus 12–17 years) have each been found to be associated with relatively poor treatment outcomes (Kendall et al., 2010).

Whether other key characteristics that distinguish children and adolescents with anxiety disorders in community populations, such as the frequency of comorbid mood and behavioral disorders, are reflected in referred samples has not been thoroughly evaluated, as young people with comorbid mood disorders or other difficulties, such as school refusal, are often excluded from treatment studies (e.g. Kendall et al., 2010). Nevertheless, higher levels of major depressive disorder (MDD) and lower levels of attention deficit disorder (ADD) have been found among adolescents compared to children with a diagnosis of overanxious disorder^1^ (OAD) (Strauss et al., 1988). There is also some suggestion that difficulties in the school environment may be more common among adolescents; when examining youth with SAD, Francis et al. (1987) found that 100% of adolescents complained of physical symptoms in school days, compared to 58–69% of children. This is consistent with the finding that over 50% of adolescents who do not regularly attend school have an anxiety disorder (Last et al., 1998, McShane et al., 2001) and is important as school refusal has serious implications for development and functioning in adolescence and adulthood (Berman et al., 2000), and may present a challenge in delivering effective treatment for anxiety disorders (Albano, 1995).

The current study compares the clinical characteristics of a consecutive series of children and adolescents referred to a routine clinical service for the treatment of anxiety disorders and builds on previous work by including a representative sample of children and young people systematically assessed for the full range of anxiety disorders, and comorbid conditions. We considered a range of factors that have been found to be associated with treatment outcome among youth with anxiety disorders, specifically, disorder subtypes (Ginsburg et al., 2011, Kerns et al., 2013), anxiety severity (Ginsburg et al., 2011, Liber et al., 2010, Southam-Gerow et al., 2001), symptoms of other common comorbid conditions (i.e. other anxiety disorders, mood and behavioral problems (Berman et al., 2000, Hudson et al., 2013), levels of school attendance (Last et al., 1998, Layne et al., 2003) and symptoms of psychopathology among caregivers (Berman et al., 2000, Cobham et al., 1998, Southam-Gerow et al., 2001). On the basis of community and clinic-based studies we hypothesized that adolescents with a primary anxiety disorder would be characterized by higher anxiety severity and more frequent social anxiety disorder, comorbid mood disorders, and irregular school attendance than children with a primary anxiety disorder. We also hypothesized that children would have more frequent SAD, and a greater number of comorbid anxiety disorders and behavior disorders than adolescents. As parental emotional difficulties have often been implicated in relation to treatment outcomes for children and adolescents with anxiety disorders (Berman et al., 2000, Cobham et al., 1998, Southam-Gerow et al., 2001), we also explored whether symptoms of anxiety, stress and depression in caregivers differed according to child/adolescent age group. Finally, as differences in clinical characteristics may be moderated by child gender (Esbjørn et al., 2010, Rao et al., 2007), we also explored the interaction between age group and gender in relation to the above variables.

## 2 Methods

### 2.1 Participants

All children and adolescents were referred by primary and secondary care services to the Berkshire Healthcare NHS Foundation Trust Child and Adolescent Mental Health Service (CAMHS) Anxiety and Depression Pathway, based at the University of Reading. This county-wide service accepts referrals for children and adolescents (up to 18 years) for the assessment and treatment of anxiety disorders. Referrals for those with a diagnosed comorbid autistic spectrum disorder (ASD) or learning disability are not accepted by this service, as they are seen within another specialist CAMHS team. In accordance with the removal of OCD and PTSD from the broad anxiety disorders category in DSM-5 (American Psychiatric Association, 2013), young people with a primary diagnosis of OCD are often referred directly to a specialist OCD service and young people with complex PTSD (e.g. where there has been abuse) would typically be referred directly to another specialist CAMHS team; therefore children and adolescents with OCD and PTSD are likely to be under-represented.

100 children between 6 and 12 years of age and 100 adolescents between 13 and 18 years of age, and their primary caregiver, were recruited. Both groups represent 100 consecutive assessments that were conducted between October 2012 and December 2013 for the children and between June 2012 and December 2013 for the adolescents. Table 1 provides demographic information for the children and adolescents in this study. Both groups contained a greater number of girls than boys, especially the adolescent group, but the gender difference between the age groups fell short of statistical significance (*χ*²(1)=3.31, *p* =.07). For the majority of children and adolescents, the primary caregiver taking part in the assessment was the child or adolescent׳s biological parent (for two children and one adolescent, the primary caregiver was a grandparent and one child had been adopted). As shown in Table 1, there were no significant differences in ethnicity, socio-economic status or caregiver gender between the age groups.

**Table 1:** Demographic information for all participants.

	**Children (*****n*****=100)**			**Adolescents (*****n*****=100)**			Statistic (children vs adolescents)
	Male (*n*=38)	Female (*n*=62)	All (*n*=100)	Male (*n*=26)	Female (*n*=74)	All (*n*=100)	
**Age in months (mean [*****SD*****], range)**	118.05 [17.44], 73–154	114.23 [21.03], 79–154	115.68 [19.74], 73–154	180.23 [2.94], 158–212	185.18 [16.88], 156–218	183.89 [16.49], 156–218	–
**Ethnicity (% White British)**	74%	84%	80%	100%	88%	91%	*χ²*(10)=15.84, *p*=.10
**Family SES (% “higher professional”)**	66%	65%	65%	65%	58%	60%	*χ²*(2)=2.79, *p*=.25
**Caregiver gender (female:male)**	33:5	58:4	91:9	23:3	70:4	93:7	*χ²(1*)=.27, *p*=.60

*Note*: SES=socio-economic status.

### 2.2 Procedure

Permission to use routine clinical information for research purposes was provided by the Clinical Audit Department of Berkshire Healthcare NHS Foundation Trust. Assessments were conducted at one time point and involved the child/adolescent and their primary caregiver being seen separately to undertake a diagnostic assessment (of the child/adolescent) and complete standardized questionnaires. All assessments were carried out by psychology graduates (assistant psychologists or trainee clinical psychologists) who received thorough training and regular supervision.

### 2.3 Measures

#### 2.3.1 Diagnoses

Children and adolescents׳ diagnoses were determined using the ADIS-C/P (Silverman and Albano, 1996). This is a structured interview, with good psychometric properties (Silverman et al., 2001), designed to assess current DSM-IV anxiety disorders. Sections on current mood, behavioral disorders and school refusal were also administered. As is standard, if the child/adolescent met symptom criteria for a diagnosis, on the basis of his/her report or that of his/her parent, the assessor assigned a Clinician Severity Rating (CSR), ranging from 0 (absent or none) to 8 (very severely disturbing/disabling); a CSR of 4 or more based on the child/adolescent and/or parent report indicated the child met criteria for diagnosis. The diagnosis with the highest CSR was classed as the primary diagnosis. Overall reliability for the assessment team was very good; reliability for the ADIS-C/P diagnosis was .92 (child report) and .82 (caregiver report) and for CSR scores was .94 (child report) and .98 (caregiver report).

#### 2.3.2 Symptom measures

The Spence Children׳s Anxiety Scale (SCAS-C/P) (Spence, 1998) assesses child/adolescent and parent-reported symptoms relating to six domains of anxiety: panic attacks/agoraphobia, separation anxiety, physical injury fears, social phobia, generalized anxiety and obsessive-compulsive symptoms. The SCAS includes 38 items to assess anxiety symptoms (and 6 positive filler items in the child version), each scored on a 4-point Likert scale, ranging from 0 (never) to 3 (always). The measure has been validated for use with children/adolescents aged from 6–18 years and both versions have good reliability, as well as discriminant and convergent validity (Nauta et al., 2004, Spence et al., 2003). Internal consistent for this scale was good to excellent (SCAS-C *α* =.82; SCAS-P *α* =.91).

The Short Mood and Feelings Questionnaire (SMFQ-C/P) (Angold et al., 1995) is a self-report measure to assess child/adolescent depression. There are versions for children/adolescents and parents to complete; both versions have 13 items and each item is scored on a 3-point scale (‘not true’, ‘sometimes’ or ‘true’). The scale has been validated with children/adolescents aged 6–17 years and has good internal reliability and discriminant validity (Angold et al., 1995). Internal consistency for the SMFQ was good to excellent (SMFQ-C *α* =.77; SMFQ-P *α* =.90).

The conduct problems subscale of the Strengths and Difficulties Questionnaire (SDQ-P) (Goodman, 1997) was administered to assess parent-reported behavioral disturbance. Five items are scored on a 3-point scale (‘not true’, ‘somewhat true’ and ‘certainly true’). The scales show acceptable internal consistencies and retest reliability (Goodman, 2001). Although there is a version for children/adolescents aged from 11–17 years old to rate themselves, the parent-report version of the SDQ was used as parents are often considered to be most reliable in reporting on children׳s externalizing symptoms (Grills and Ollendick, 2003). Although internal consistency was poor (SDQ-P conduct problems *α* =.514), this is likely to reflect the relatively low number of items in the subscale.

#### 2.3.3 Caregivers׳ symptoms of anxiety, depression and stress

Caregivers׳ own symptoms of psychological functioning were assessed using the short-version of the Depression Anxiety Stress Scales (DASS) (Lovibond and Lovibond, 1995). This 21-item, self-report measure has three scales relating to symptoms of anxiety, stress and depression. Each scale consists of 7 items and items are scored using a scale of 0 to 3, where 0 ‘did not apply to me at all’ and 3 ‘applied to me very much or most of the time’. The DASS-21 has been used with non-clinical and clinical populations and the scales show good internal consistency and concurrent validity (Antony et al., 1998). Internal consistency for the DASS was good to excellent (DASS-D *α* =.92; DASS-A *α* =.85; DASS-S *α* =.88).

### 2.4 Data analysis

Main effects of age group and gender and the interaction of age group *x* gender in predicting clinical characteristics were examined using binary logistic regression for dichotomous variables, and factorial analysis of variance for continuous variables. However, there were no significant effects of gender or gender *x* age group interactions and so simple tests of association with age group are reported below (independent *t*-tests and Pearson׳s chi-square tests) for ease of interpretation. For non-Normally distributed data, differences were explored using non-parametric and parametric tests; as results did not differ, analyses using parametric tests will be reported. All tests were two-tailed. Because of small cell numbers in some cases, statistical analyses were only run for the most common disorders (SAD, GAD, social anxiety disorder and specific phobias). Descriptive data are given for all disorders and are presented in Table 2, Table 3.

**Table 2:** Child/adolescent primary disorder and Anxiety Disorder Severity Ratings as measured by the ADIS-C/P.

		**Children**			**Adolescents**			**Statistic**
		Male (*n*=38)	Female (*n*=62)	All (*n*=100)	Male (*n*=26)	Female (*n*=74)	All (*n*=100)	(children vs adolescents)
**Anxiety disorders**	SAD	5 (13%)	14 (23%)	19	2 (8%)	2 (3%)	4	*χ*²(1)=11.33, *p*=.001
	Social anxiety disorder	6 (16%)	11 (18%)	17	8 (33%)	21 (28%)	29	*χ²*(1)=4.31, *p*=.04
	GAD	8 (21%)	18 (29%)	26	6 (25%)	13 (18%)	19	*χ²*(1)=1.49, *p*=.22
	Specific phobia	4 (11%)	8 (13%)	12	3 (12%)	15 (20%)	18	*χ²*(1)=1.46, *p*=.23
	PD w/o agoraphobia	0	0	0	1 (4%)	2 (3%)	3	–
	PD with agoraphobia	0	0	0	3 (12%)	4 (5%)	7	–
	Agoraphobia w/o PD	1 (3%)	1 (2%)	2	1 (4%)	0	1	–
	PTSD	0	0	0	0	0	0	–
	OCD	2 (5%)	1 (2%)	3	0	3 (4%)	3	–
	ADNOS	2 (5%)	3 (5%)	5	0	0	0	–
**Total anxiety disorders**		28 (74%)	56 (90%)	84	24 (92%)	60 (81%)	84	–
**CSR for anxiety**	4 (moderate)	9 (32%)	7 (13%)	16	1 (4%)	4 (5%)	5	–
**disorder**	5 (moderate)	5 (18%)	22 (39%)	27	9 (38%)	20 (33%)	29	–
	6 (severe)	13 (46%)	24 (43%)	37	9 (38%)	21 (35%)	30	–
	7 (severe)	1 (4%)	3 (5%)	4	5 (21%)	15 (25%)	20	–
	8 (very severe)	0	0	0	0	0	0	–
**CSR (mean [*****SD*****])**		5.21 [.96]	5.41 [.78]	5.35 [.84]	5.75 [.85]	5.78 [.90]	5.77 [.88]	*t*(166)= −3.22, *p* <.01
**Comorbid anxiety disorders (mean [*****SD*****])**		0.61 [.96]	1.20 [1.20]	1.00 [1.15]	0.83 [.82]	0.82 [.79]	0.82 [.79]	*t*(166)=1.17, *p* =.24
**Mood disorder**	MDD	0	1 (2%)	1	1 (4%)	7 (9%)	8	–
	Dysthymic disorder	0	0	0	0	0	0	–
**Other**	ODD	4 (11%)	0	4	0	1 (1%)	1	–
	Conduct disorder	0	0	0	0	0	0	–
	ADHD	1 (3%)	0	1	0	0	0	–
**No disorder**		5 (13%)	5 (8%)	10	1 (4%)	6 (8%)	7	*χ²*(1)=.58, *p*=.45

*Note*: CSR= Clinical Severity Rating, SAD=separation anxiety disorder, PD=panic disorder, GAD=generalized anxiety disorder, PTSD=post-traumatic stress disorder, OCD=obsessive-compulsive disorder, ADNOS=anxiety disorder not otherwise specified, MDD=major depressive disorder, ODD=oppositional defiant disorder, ADHD=attention deficit hyperactivity disorder.

**Table 3:** Occurrence of all DSM-IV disorders and school refusal in children/adolescents with a primary anxiety disorder, as measured by the ADIS-C/P.

		**Children**			**Adolescents**			**Statistic**
		Male (*n*=28)	Female (*n*=56)	All (*n*=84)	Male (*n*=24)	Female (*n*=60)	All (*n*=84)	(children vs adolescents)
**Anxiety disorder**	SAD	9 (32%)	28 (50%)	37 (44%)	5 (21%)	10 (17%)	15 (18%)	*χ²*(1)=13.48, *p<*.001
	Social anxiety disorder	9 (32%)	29 (52%)	38 (45%)	14 (58%)	30 (50%)	44 (52%)	*χ²*(1)=0.86, *p*=.35
	GAD	12 (43%)	29 (52%)	41 (49%)	12 (50%)	34 (57%)	46 (55%)	*χ²*(1)=0.60, *p*=.44
	Specific phobia	8 (29%)	22 (39%)	30 (36%)	8 (33%)	18 (30%)	26 (31%)	*χ²*(1)=0.43, *p*=.51
	PD w/o agoraphobia	1 (4%)	0	1 (1%)	1 (4%)	4 (7%)	5 (6%)	–
	PD with agoraphobia	0	0	0	3 (13%)	4 (7%)	7 (8%)	–
	Agoraphobia w/o PD	1 (4%)	1 (2%)	2 (2%)	1 (4%)	0	1 (1%)	–
	PTSD	0	0	0	0	1 (2%)	1 (1%)	–
	OCD	2 (7%)	3 (5%)	5 (6%)	0	7 (12%)	7 (8%)	–
	ADNOS	2 (7%)	3 (5%)	5 (6%)	0	0	0	–
**Total anxiety disorders**		44	123	167	44	109	153	
**Anxiety disorders (mean [***SD***])**		1.61 [.96]	2.20 [1.20]	2.00 [1.15]	1.83 [.82]	1.82 [.79]	1.82 [.79]	*t*(166)=1.17, *p*=.24
**CSR for anxiety**	4 (moderate)	19 (43%)	48 (39%)	67 (40%)	12 (27%)	18 (16%)	30 (20%)	–
**disorder**	5 (moderate)	8 (18%)	41 (33%)	49 (29%)	15 (34%)	47 (43%)	62 (40%)	–
	6 (severe)	15 (34%)	31 (25%)	46 (28%)	12 (27%)	29 (27%)	41 (27%)	–
	7 (severe)	2 (5%)	3 (3%)	5 (3%)	5 (12%)	15 (14%)	20 (13%)	–
	8 (very severe)	0	0	0	0	0	0	–
**CSR (mean [*****SD*****])**		5.00 (.99)	4.91 (.86)	4.93 (.89)	5.23 (.99)	5.38 (.92)	5.33 (.94)	*t*(318)= −3.90, *p*<.001
**Mood disorder**	MDD	1 (4%)	0	1 (1%)	3 (13%)	5 (8%)	8 (10%)	–
	Dysthymic disorder	2 (7%)	3 (5%)	5 (6%)	5 (21%)	11 (18%)	16 (19%)	–
	Total mood disorder	3	3	6	8	16	24	*χ²*(1)=13.15, *p*<.001
**Other**	Selective mutism	0	1 (2%)	1 (1%)	0	0	0	–
	ODD	1 (4%)	4 (7%)	5 (6%)	1 (4%)	4 (7%)	5 (6%)	–
	Conduct disorder	0	0	0	0	0	0	–
	ADHD	2 (7%)	3 (5%)	5 (6%)	1 (4%)	2 (3%)	3 (4%)	–
**School refusal**		1 (20%)	5 (9%)	6 (7%)	5 (21%)	10 (17%)	15 (18%)	*χ²*(1)=4.41, *p*=.04

*Note*: CSR=Clinical Severity Rating, SAD=separation anxiety disorder, PD=panic disorder, GAD=generalized anxiety disorder, PTSD=post-traumatic stress disorder, OCD=obsessive-compulsive disorder, ADNOS=anxiety disorder not otherwise specified, MDD=major depressive disorder, ODD=oppositional defiant disorder, ADHD=attention deficit hyperactivity disorder.

## 3 Results

### 3.1 Primary disorder

Eighty-four percent of both the child and adolescent groups met criteria for an anxiety disorder as the primary diagnosis on the ADIS (see Table 2). Where this was not the case, 6% of children and 9% of adolescents had another primary disorder (of the children, 4% had ODD, 1% had ADHD and 1% had MDD; of the adolescents, 8% had MDD and 1% had ODD) and 10% of children and 7% of adolescents had a sub-clinical level of symptoms and so did not meet criteria for any disorder. The following analyses are conducted with children and adolescents with an anxiety disorder as the primary diagnosis.

### 3.2 Anxiety disorders

The frequency of primary DSM-IV diagnoses for both groups are shown in Table 2, and the overall frequency of anxiety disorders are shown in Table 3. Children were significantly more likely than adolescents to be diagnosed with SAD as the primary disorder or anywhere in the diagnostic profile; the odds of being diagnosed with SAD as the primary disorder were 5.85 times higher for children than adolescents (95% *CI* 1.89, 18.04) and the odds of being diagnosed with SAD overall were 3.62 times higher for children than adolescents (95% *CI* 1.79, 7.33). Adolescents were significantly more likely than children to be diagnosed with social anxiety disorder as the primary disorder, with the odds 2.08 higher for adolescents than children (95% *CI* 1.04, 4.17). The frequency of social anxiety disorder overall, however, was not significantly higher in the adolescents than the children. The differences between age groups for the frequency of GAD and specific phobias were not significant, either as the primary or overall diagnosis. The mean number of comorbid anxiety disorders was not significantly different for children (mean=1.00, *SD*=1.15) and adolescents (mean=.82, *SD*=.79; *t*(166)=1.17, *p* =.24), and the same pattern was found when looking at the number of comorbid disorders associated with each of the most common primary anxiety disorders (SAD, GAD, social anxiety disorder and specific phobias). Clinician-rated severity levels for both the primary anxiety disorder and for all anxiety disorders were significantly higher for adolescents than children, with effect sizes in the small to medium range (primary anxiety disorder CSR *d* =.49; all anxiety disorder CSR *d* =.44).

### 3.3 Comorbid mood disorders

Adolescents with a primary anxiety disorder were significantly more likely to be diagnosed with a comorbid mood disorder (MDD or dysthymic disorder) than children, with the odds 5.20 higher (95% *CI* 2.00, 13.52) for adolescents than for children (see Table 3).

### 3.4 School refusal

Significantly more adolescents than children with a primary anxiety disorder were not regularly attending school; the odds were 2.83 higher (95% *CI* 1.04, 7.69) for adolescents than for children (see Table 3).

### 3.5 Symptom measures

Means and standard deviations for all symptom measures can be found in Table 4. Adolescents with a primary anxiety disorder scored significantly higher than children with a primary anxiety disorder on both self-reported symptoms of anxiety and depression, with small to medium effect sizes (SCAS-C *d* =.32; SMFQ-C *d* =.37). Differences between groups were not significant for caregiver-reported child/adolescent symptoms of anxiety, depression or behavioral disturbance or for caregiver symptoms of anxiety, stress and depression.

**Table 4:** Summary of means, standard deviations and statistics for self-report data for children/adolescents with a primary anxiety disorder.

		**Children**			**Adolescents**			**Statistic**
		Male (_max_*n*=28)	Female (_max_*n*=56)	All (_max_*n*=84)	Male (_max_*n*=24)	Female (_max_*n*=60)	All (_max_*n*=84)	(children vs adolescents)
**Child/adolescent**	SCAS total	35.14 (15.20)	39.26 (17.38)	37.84 (16.68)	38.95 (15.32)	44.43 (14.92)	42.97 (15.12)	*t*(158)= −2.04, *p*=.04
**Report**	SMFQ total	6.29 (4.37)	7.19 (5.56)	6.88 (5.17)	8.39 (6.13)	9.77 (8.58)	9.38 (7.94)	*t*(158) = –2.36, *p*=.02
**Caregiver Report**	SCAS	30.79 (15.28)	36.63 (14.01)	34.59 (14.64)	40.65 (21.80)	37.44 (18.96)	38.34 (19.71)	*t*(160)= –1.37, *p*=.17
	SMFQ	6.89 (5.77)	7.16 (5.91)	7.07 (5.83)	9.48 (7.88)	8.55 (6.78)	8.81 (7.07)	*t*(161) = –1.72, *p*=.09
	SDQ conduct	2.39 (1.95)	2.11 (1.94)	2.20 (1.94)	1.57 (1.38)	1.98 (1.75)	1.86 (1.65)	*t*(159)=1.22, *p*=.23
	DASS-Dep	5.26 (6.33)	7.69 (8.99)	6.85 (8.21)	7.50 (9.46)	6.75 (8.02)	6.96 (8.41)	*t*(159)= –0.09, *p*=.93
	DASS-Anx	4.30 (4.63)	5.92 (7.90)	5.35 (6.94)	4.00 (4.49)	6.21 (7.60)	5.56 (6.89)	*t*(157)= –1.19, *p*=.85
	DASS-Stress	9.93 (6.00)	13.63 (7.97)	12.37 (7.53)	11.58 (8.00)	11.90 (8.72)	11.81 (8.48)	*t*(163)=0.45, *p*=.66

*Note*: Where data was missing, this was less than 10% of the dataset. DASS=Depression Anxiety and Stress Scales, Dep=Depression, Anx=Anxiety, SCAS=Spence Child Anxiety Scale, SDQ=Strengths and Difficulties Questionnaire, SMFQ=Short Moods and Feelings Questionnaire.

## 4 Discussion

Among a consecutive series of referrals for treatment for anxiety disorders, the majority of children and adolescents (84%) were appropriate, meeting diagnostic criteria for a primary anxiety disorder. Where this was not the case, around half the children and adolescents did not meet clinical thresholds, and the other half had either a primary mood (particularly among adolescents) or behavioral (particularly among children) disorder. Consistent with the hypotheses, adolescents with a primary anxiety disorder were significantly more likely than children to (i) be rated by a clinician as having more severe anxiety for both the primary anxiety disorder and anxiety disorders overall and rate themselves as having higher levels of anxiety symptoms, (ii) be diagnosed with social anxiety disorder as the primary disorder, (iii) be diagnosed with a comorbid mood disorder and rate themselves as having higher levels of depressive symptoms, and (iv) have irregular school attendance. In addition, more adolescents than children were diagnosed with panic disorder and/or agoraphobia (but due to the small numbers in children, this difference was not tested statistically). Also consistent with the hypotheses, children with a primary anxiety disorder were significantly more likely to be diagnosed with SAD than adolescents as either the primary anxiety disorder or anywhere in the diagnostic profile. Contrary to hypotheses, there were no significant differences in the frequency of behavioral disorders or symptoms of conduct problems, however there were relatively low levels of comorbid behavioral disturbance in both groups. Although there were a greater number of girls than boys in both age groups, gender was not significantly associated with any of the clinical characteristics either on its own or in an interaction with age. We also did not find significant differences between the age groups on the frequency of GAD and specific phobias (as primary disorders or overall), the frequency of social anxiety disorder overall, frequency of comorbid anxiety disorders or for symptoms of psychopathology among primary caregivers.

Consistent with the results of community studies (Cohen et al., 1993, Compton et al., 2000, Copeland et al., 2014, Costello, 2003, Costello et al., 2003, Ford et al., 2003), in this clinical population we found significantly lower rates of SAD in adolescents compared to children, a higher frequency of panic disorder and agoraphobia and social anxiety disorder as a primary diagnosis. However, unlike community studies (e.g. Copeland et al., 2014, Costello, 2003), when we considered diagnoses of social anxiety disorder anywhere in the diagnostic spectrum, we did not find significant differences according to age. In other words, social anxiety disorder is not more common among adolescents compared to children, but it was more likely to be causing the greatest disturbance. Whether this is due to increasing social demands that may come with adolescence requires further exploration.

We found that adolescents׳ anxiety symptoms were rated as more severe than the children, replicating findings from two other studies of clinical populations (Kendall et al., 2010, Strauss et al., 1988) and that adolescents experienced higher levels of comorbid depressive disorders, also consistent with findings from a clinical population (Strauss et al., 1988). This is of particular note in light of Wittchen et al.׳s(2003) finding that more severe anxiety disorders were associated with an increased risk of subsequent depression. This may reflect more pervasive interference with the ability to undertake important educational, social and leisure activities and achieve crucial milestones among adolescents. In addition, difficulties regularly attending school are commonly associated with anxiety (Francis et al., 1987, Last et al., 1998); we found that this was particularly the case among adolescents. It is of interest, however, that greater levels of symptom severity, mood disorders and difficulties attending school are not mirrored by increased symptoms of depression, anxiety and stress among caregivers of adolescents.

### 4.1 Strengths

The strengths of this research are that the young people come from a routine clinical service for young people with anxiety and depression and unlike existing studies (e.g. Francis et al., 1987, Kendall et al., 2010, Rao et al., 2007, Strauss et al., 1988) include young people with the full range of anxiety disorders, comorbid mood disorders, school refusal and previous non-response to treatment. As such, results are likely to be broadly generalizable to other children and adolescents within standard clinical care settings. A further strength is that extensive assessments were undertaken by trained and reliable assessors, using standardized clinical interviews and well-validated self-report measures, with the young people and their caregivers assessed separately to increase reliability (Costello, 1989).

### 4.2 Limitations

Nevertheless, there are limitations to the study, in that age was considered within two categories, determined on the basis that childhood and adolescence can be seen as distinct, developmental periods (Erikson, 1968); in reality changes would be unlikely to occur in such a discrete manner, and future studies should aim to look at still narrower age bands. In addition, children and adolescents were from a predominantly White British ethnic background and from relatively high socio-economic backgrounds. The sample did not include children or adolescents identified with ASD and young people with OCD and PTSD may also be under-represented.

### 4.3 Implications for treatment

The finding that children and adolescents referred for treatment for anxiety disorders have distinct clinical characteristics has clear implications for treatment and suggests that adapting treatments designed for children to make the materials more ‘adolescent-friendly’ is unlikely to sufficiently meet the needs of adolescents. Indeed, all of the characteristics which distinguished adolescents from children with a primary anxiety disorder have been found to be associated with reduced remission following treatment (Ginsburg et al., 2011). It is of importance, therefore, that programs are designed and delivered that adequately address these characteristics in the treatment of anxiety disorders in adolescence.

## Role of funding source

This research was supported by MRC Clinical Research Training Fellowship (G1002011), awarded to Dr. Waite.

## Conflict of interest

None.

## Footnotes

1 The diagnostic category of OAD (American Psychiatric Association, 1987) was characterized by excessive worries about multiple future and past events, and was replaced by Generalized Anxiety Disorder (GAD) in DSM-IV (American Psychiatric Association, 1994).
